# Guest editorial: Extensive use of monosodium glutamate: a threat to public health?

**DOI:** 10.17179/excli2018-1092a

**Published:** 2018-10-02

**Authors:** Kamal Niaz, Elizabeta Zaplatic, Jonathan Spoor

**Affiliations:** 1 Faculty of Bioscience and Agri-Food and Environmental Technology, University of Teramo-64100, Italy; 2 Erasmus University Medical Centre, Erasmus University Rotterdam, Rotterdam, the Netherlands

## ⁯

Corrigendum: A previous version of this article contained a wrong affiliation of Kamal Niaz. Please refer only to these revised affiliations which are corrected accordingly. 

